# Agenesis and Hypomyelination of Corpus Callosum in Mice Lacking Nsun5, an RNA Methyltransferase

**DOI:** 10.3390/cells8060552

**Published:** 2019-06-06

**Authors:** Zihao Yuan, Peipei Chen, Tingting Zhang, Bin Shen, Ling Chen

**Affiliations:** 1 State Key Laboratory of Reproductive Medicine, Department of Physiology, Nanjing Medical University, Nanjing 211166, China; 15250963556@yeah.net (Z.Y.); 15720803023@163.com (P.C.); ztt19921004@163.com (T.Z.); 2 Department of Physiology, Nanjing Medical University, Nanjing 211166, China

**Keywords:** Nsun5, Williams-Beuren syndrome (WBS), corpus callosum (CC), oligodendrocyte (OL), myelination

## Abstract

Williams-Beuren syndrome (WBS) is caused by microdeletions of 28 genes and is characterized by cognitive disorder and hypotrophic corpus callosum (CC). *Nsun5* gene, which encodes cytosine-5 RNA methyltransferase, is located in the deletion loci of WBS. We have reported that single-gene knockout of *Nsun5* (*Nsun5*-KO) in mice impairs spatial cognition. Herein, we report that postnatal day (PND) 60 *Nsun5*-KO mice showed the volumetric reduction of CC with a decline in the number of myelinated axons and loose myelin sheath. Nsun5 was highly expressed in callosal oligodendrocyte precursor cells (OPCs) and oligodendrocytes (OLs) from PND7 to PND28. The numbers of OPCs and OLs in CC of PND7-28 *Nsun5*-KO mice were significantly reduced compared to wild-type littermates. Immunohistochemistry and Western blot analyses of myelin basic protein (MBP) showed the hypomyelination in the CC of PND28 *Nsun5*-KO mice. The *Nsun5* deletion suppressed the proliferation of OPCs but did not affect transition of radial glial cells into OPCs or cell cycle exit of OPCs. The protein levels, rather than transcriptional levels, of CDK1, CDK2 and Cdc42 in the CC of PND7 and PND14 *Nsun5*-KO mice were reduced. These findings point to the involvement of *Nsun5* deletion in agenesis of CC observed in WBS.

## 1. Introduction

Williams-Beuren syndrome (WBS; MIM 194,050) is a rare (7.5–10/100,000) and complex neuro-developmental disorder with multisystemic manifestations [1]. WBS is caused by 26 to 28 contiguous gene deletions [2] on human chromosome 7q11.23 [1]. These microdeletions have been detected in 90–99% of individuals with WBS [3].

The corpus callosum (CC) is the largest interhemispheric commissure known to modulate cerebral specialization and interhemispheric communication. During brain development, midline structures, including the CC, are the most vulnerable to the influence of complex mechanical and genetic factors [4]. Previous studies that analyzed the CC in WBS reported morphologic abnormalities and volumetric reductions. Luder et al. [5] found a significantly thinner callosal region in patients with WBS. In close agreement with the observation, the CC in WBS patients has been observed to be shorter and less curved [6]. Moreover, callosal midline lengths were found to be reduced and the callosal bending angles were enlarged [7,8]. A recent study reported the neuroradiological features in the brains of 12 WBS patients and showed several structural abnormalities of the central nervous system, for example, a hypotrophic CC and hypoplastic temporal lobes [9]. A shorter, less curved and thinner posterior callosal region might be associated with the unique cognitive and behavioral profile of WBS patients [5]. Thus, the abnormal shape of CC is an attractive candidate for exploring the pathological mechanisms underlying the cognitive aspects associated with WBS.

The *Nsun5* gene, which encodes a cytosine-5 RNA methyltransferase, is deleted in WBS [1,10]. Nsun5 is deleted in about 95% patients with WBS [11]. Nsun5 can directly methylate cytosine 2278 of 25S ribosomal RNA (rRNA) [11,12]. The lack of this methylation has been demonstrated to alter the structural conformation of the ribosome and to reduce the translational fidelity [13]. We have recently reported that Nsun5 was selectively expressed in the oligodendrocyte precursor cells (OPCs) of adult hippocampal gray matter [14]. The single-gene *Nsun5* knockout (*Nsun5*-KO) in mice impairs the development of OPCs [14].

Although the majority OPCs appear in early neonatal rodent brains, the maturation and myelination of oligodendrocytes (OLs) occur largely between postnatal day (PND) 7 and PND28 [15]. During CC development, OPCs proliferate and differentiate from PND7 to become mature OLs [16]. OPCs exit the cell cycle, become postmitotic OLs and further mature into myelinating OLs. The formation of myelin provides essential trophic support for CC axonal development during the developmental growth windows and mediates the fast conduction of neuronal information [17,18]. The Nsun5 transcript is enriched in the developing mouse brain. Nsun5 deficiency has been found to reduce the proliferation of OPCs in the adult hippocampus [14]. Therefore, it is of great interest to investigate whether the Nsun5 deficiency affects the development of CC.

To this end, we employed *Nsun5*-KO mice and observed their midline structures of the CC and axonal myelination. To explore the underlying molecular mechanisms, we further examined the proliferation and differentiation of OPCs and the maturation and myelination of OLs during CC development of *Nsun5*-KO mice. The results indicate that Nsun5 is required for the development of the CC by regulating the proliferation of OPCs and myelination of OLs. This finding points to the Nsun5 deficiency is associated with the agenesis of CC observed in WBS patients.

## 2. Materials and Methods

### 2.1. Generation and Identification of Nsun5-null Mice

All animals were treated according to the guidelines of Animal Care by the Institutional Animal Care and Ethical Committee of Nanjing Medical University (No. 2014-153). The generation of *Nsun5*-KO mice was performed by CRISPR/Cas9 genome editing. The in vitro transcription and microinjection of CRISPR/Cas9 has been previously described [19]. Two sgRNAs were designed to target exon 3 of the *Nsun5* gene. The oligos for the generation of sgRNA expression plasmids were annealed and cloned into the BsaI sites of pUC57-sgRNA (Addgene 51,132). Oligo sequences are sgRNA1-sense: TAGGCCCAGCAGAGCCTTCCAT; sgRNA1-antisense: AAACATGGAAGGCTCTGCTGGG; sgRNA2-sense: TAGGCTGAGCTGGCCCGACTCA; sgRNA2-antisense: AAACTGAGTCGGGCCAGCTCAG. The *Nsun5*-KO mice were backcrossed with C57BL/6 background mice for over 10 generations. The homozygous *Nsun5*-KO mice used in the present study were obtained by mating between heterozygous *Nsun5* mice. Genotyping was determined by polymerase chain reaction (PCR) examination using the genomic DNA obtained from tail biopsies [14]. The genotyping primers were: 5′-CTGTCCAGGTGCTAGTGTATG-3′ and 5′-GGTCCTCATTTCGGCTCAC-3′. The mice were maintained under constant conditions (temperature of 23 ± 2 °C, humidity of 55 ± 5% and a 12:12-h light/dark cycle) with free access to food and water.

Postnatal day (PND) 3 WT mice (*n* = 12), PND7 WT mice (*n* = 36) and *Nsun5*-KO mice (*n* = 36), PND14 WT mice (*n* = 24) and *Nsun5*-KO mice (*n* = 24), PND28 WT mice (*n* = 18) and *Nsun5*-KO mice (*n* = 18), PND60 WT mice (*n* = 24) and *Nsun5*-KO mice (*n* = 24) were used in the preset study. The mice were randomly divided into 4 experimental groups. In the first group, the samples of CC were harvested from PND3 (*n* = 6) WT mice, PND7 (*n* = 12), PND14 (*n* = 12), PND28 (*n* = 6) and PND60 (*n* = 6) WT mice and *Nsun5*-KO mice, to examine the expression of Nsun5, MBP, CDK1, CDK2, Cdc42 and RhoA by real-time PCR and Western blot analysis. In the second group, PND60 (*n* = 18) WT mice and *Nsun5*-KO mice were required for Luxol fast blue (LFB) staining of mid-sagittal CC (*n* = 6) or coronal CC sections (*n* = 6) and ultrastructural examination of myelin (*n* = 6). In the third group, PND3 (*n* = 6) WT mice, PND7 (*n* = 12), PND14 (*n* = 12) and PND28 (*n* = 12) WT mice and *Nsun5*-KO mice were used in experiments of immunohistochemistry. In the fourth group, PND7 (*n* = 12) WT mice and *Nsun5*-KO mice were treated with the injection of BrdU to label 2 h (*n* = 6) and 24 h (*n* = 6) proliferating cells, respectively.

### 2.2. Antibodies

The following antibodies were used for immunohistochemistry or western blot analyses: rabbit anti-Nsun5 (15449-1-AP, Proteintech Group Inc., Wuhan, China; Western blot,1:300; IF,1:100), rat anti-MBP (MAB386, Millipore, Billerica, MA, USA; Western blot,1:500; IF,1:600), rabbit anti-PDGFRα (ab203491, Abcam, Cambridge, UK, 1:500), mouse anti-BrdU (MAB4072, Millipore, 1:1000), rabbit anti-NG2 (AB5320, Millipore, 1:200), rabbit anti-BLBP (ab32423, Abcam, 1:1000), mouse anti-CC1 (OP80, Millipore, 1:200), rabbit anti-Ki67 (ab16667, Abcam, 1:500), mouse anti-olig2 (MABN50, Millipore, 1:300), rat anti-NG2 (MAB6689-SP, Millipore, 1:400), rabbit anti-CDK2 (ab32147, Abcam, 1:5000), rabbit anti-CDK1 (19532-1-AP, Proteintech, 1:1000), rabbit anti-cleaved caspase-3 (ab2302, Abcam; 1:300) and rabbit anti-RhoA (10749-1-AP, Proteintech, 1:1000). The secondary antibodies-donkey anti-mouse or anti-rat-conjugated to either Alexa Fluor 488 (Jackson ImmunoResearch, West Grove, PA, USA, 1:500) or 555 (Jackson ImmunoResearch; 1:500) and the biotinylated goat anti-mouse secondary antibody (Santa Cruz Biotechnology, Santa Cruz, CA, USA, 1:200) was directed against the primary IgG antibody species.

### 2.3. Histology Analyses

The mice were injected with pentobarbital (50 mg/kg) and were transcardially perfused with cold PBS followed by 4% paraformaldehyde. For the analysis of OPC proliferation, mice were injected intraperitoneally (i.p.) with the thymidine analogue BrdU (Sigma-Aldrich, St. Louis, MO, USA) at a concentration of 50 mg/kg body weight [20]. Two hours later, the perfused brains were removed. For examination of cell cycle exit, the mice were injected with BrdU (50 mg/kg, i.p.) and were sacrificed 24 h later [21].

The brains were removed, and dissected brains were post-fixed in 4% paraformaldehyde overnight at 4 °C. For frozen sections, the brains were transferred into 15% and 30% sucrose. After the brains completely sank to the bottom in 30% sucrose, the sagittal sections (30 μm) or coronal sections (10 μm) were cut using a cryostat (Leica CM3050S; Leica Microsystems, Heidelberg, Germany). The coronal sections were cut continuously from the rostral to caudal CC and then were divided equally into 7 parts.

Luxol Fast Blue staining: The mid-sagittal CC sections and coronal CC sections were immersed in 0.1% Luxol Fast Blue solution at 37 °C for 12–16 h and then in 95% ethanol for 5 min. These sections were incubated in 0.05% lithium carbonate solution for 1 min and then were washed with 70% ethanol and distilled water. After crystal violet counterstaining, the sections were sealed for microscopic observation.

Immunohistochemistry: The coronal sections were incubated in 3% hydrogen peroxide for 30 min and were subsequently treated with 1% bovine serum albumin (BSA, Sigma Chemical Co.) for 60 min to block nonspecific binding. After blocking, sections were incubated with the primary antibodies overnight at 4 °C. The primary antibodies were visualized by incubating the sections with the appropriate fluorophore-conjugated secondary antibodies or biotinylated IgG antibodies for 2 h at room temperature. The immunoreactivities were visualized using an avidin biotin horseradish peroxidase complex (Vector Laboratories, Burlingame, CA, USA). Sections were counterstained with DAPI (1:1000; Sigma) and mounted with mounting medium (Vector Laboratories). The immunoreactivities were visualized using fluorescence microscopy (DP70; Olympus, Tokyo, Japan) or a conventional light microscope (DP70; Olympus). For double immunofluorescence staining, the sections were simultaneously incubated with two primary antibodies that were developed in different species and diluted in 1% BSA, overnight at 4te. Primary antibodies were detected with appropriate secondary antibodies for 2 h at room temperature. Images of stained sections were observed using a fluorescence microscope.

Transmission electron microscopy (TEM): The brains were immersed in 2.5% glutaraldehyde and 2% paraformaldehyde. After overnight fixation, a block (1 × 1 × 2 mm^3^) was dissected from the CC at the level of the anterior-dorsal hippocampus under a Leica stereomicroscope (MZ 6; Leica Pte, Leica, Heidelberg, Germany). The samples were post-fixed in 1% osmium tetroxide and then dehydrated and embedded in Epon-Araldite. Ultrathin sections were cut and stained with uranyl acetate and lead citrate. The samples were observed and photographed using a JEM-1400 Transmission Electron Microscope (JEOL USA, Peabody, MA, USA).

**Figure 1:**
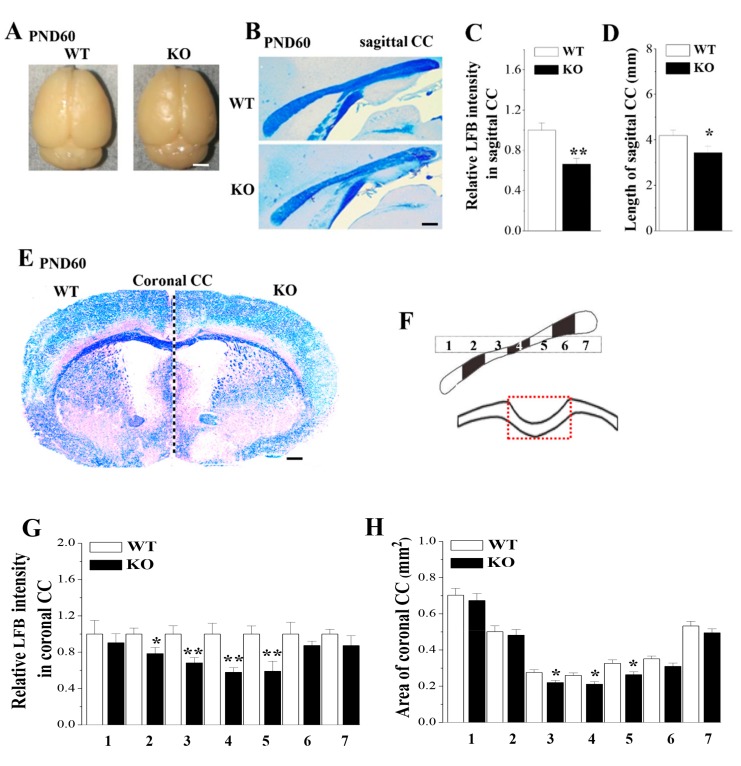
Loss of Nsun5 causes corpus callosum (CC) agenesis with hypomyelination. (**A**) Pictures of entire brain of PND60 WT mice (WT) and *Nsun5*-KO mice (KO). Scale bar = 0.25 cm. (**B**) Representative images of mid-sagittal CC stained with Luxol Fast Blue (LFB). Scale bar = 100 μm. (**C, D**) Bar graphs show the intensity of LFB staining and lengths of mid-sagittal CC in WT mice and *Nsun5*-KO mice. * *p* < 0.05, ** *p* < 0.01 vs. WT (*n* = 6). Error bars represent the mean ± SEM. (**E**) Representative images of the coronal CC of WT mice (left side) and *Nsun5*-KO mice (right side). Scale bar = 500 μm. (**F**) Schematic diagram of coronal sections from rostral to caudal CC divided equally into 7 segments (upper panel) and measured region in coronal CC (red dashed box). (**G**, **H**) Bar graphs represent the intensity of LFB staining and areas of coronal CC obtained from 7 segments, respectively. * *p* < 0.05, ** *p* < 0.01 vs. WT (one-way ANOVA, *n* = 6).

Morphometric analysis and quantization: (1) The areas of mid-sagittal CC (3 sections/mouse) and coronal CC (2 sections per segment, total 7 segments/mouse; Figure 1E) were measured using digital photographs with a semiautomated image analysis system (ImagePro Plus V. 4.5, CyberneticsMedia, Silver Spring, MD, USA). The measurements were repeated 3 times for each sample to obtain an average value; (2) The mid-sagittal CC and coronal CC sections stained with LFB were scanned using a Leica scanner. Images from at least 6 sections were collected and quantitative image analysis was performed using ImageJ software package (National Institutes of Health) as described previously [22]. The intensity of LFB staining in each experimental group were normalized to controls and presented as bar graphs; (3) At least 6 sections for each mouse and each experimental condition were analyzed and counted. An average of 6 sections was quantified to obtain the number of positive cells per mouse. Cell counting was performed blindly; (4) The number of Ki67-/BrdU+ cells (cell cycle) was divided by total number of BrdU+ cells in the CC to obtain the cell-cycle exit index [21]; and (5) The myelinated axons were calculated from at least 10 sections for each mouse. The thickness of myelination was quantified by G ratio (the numerical ratio of the axonal diameter divided by the diameter of the myelinated fiber) [23]. Approximately 120 myelinated axons per block were randomly selected for a detailed morphometric analysis using ImageJ software (http://rsb.info.nih.gov/ij/). The cases were coded to facilitate blind quantification.

### 2.4. Western Blot Analysis

Each CC was dissected under a Leica stereomicroscope (MZ 6; Leica Pte) and was sonicated in 200 μL of Tris buffer (pH 7.4) containing 10% sucrose, phosphatase inhibitors and protease inhibitors (Complete; Roche Diagnostics). The protein concentrations were quantified by a Bio-Rad Protein Assay Kit (Bio-Rad, Rockford, IL, USA) according to the manufacturer’s protocol. Equal amounts of proteins were separated by SDS-polyacrylamide gel electrophoresis and were transferred to PVDF membranes. The membranes were blocked with 5% nonfat milk in Tris-buffered saline/Tween-20 (TBS-T) and were then incubated with antibodies against CDK1, CDK2, Cdc42 and RhoA at 4 °C overnight. The appropriate horseradish peroxidase (HRP)-conjugated secondary antibodies were incubated with the membranes for 1 h at room temperature. The signals were visualized using an enhanced chemiluminescence detection kit (ECL, Millipore). Following visualization, the blots were stripped with stripping buffer for 15 min and were then incubated with the antibodies against the protein at 4 °C overnight. The Western blot bands were scanned and analyzed using the ImageJ software package.

### 2.5. Reverse Transcription-Polymerase Chain Reaction (RT-PCR)

The CC was immediately transferred to TRIzol Reagent (Invitrogen, Camarillo, CA, USA) and was processed for total RNA isolation according to the manufacturer’s protocol and was quantified by spectrophotometry. Then, the RNA was reverse-transcribed into cDNAs using a Prime Script RT reagent kit (Takara, Japan) for quantitative PCR (ABI Step One Plus, Foster City, CA, USA) in the presence of a fluorescent dye (SYBR Green I; Takara, Japan). The relative expression of the genes was calculated using the 2^−ΔΔct^ method with normalization to the *GAPDH* expression level. The primer sequences were designed based on published sequences of mouse genes listed in Table 1 [24,25].

### 2.6. Data Analysis/Statistics

The data were processed with Origin 9.1 software (Origin Lab Corp., Northampton, MA, USA). The group’s data were expressed as the mean plus or minus the standard error of the mean (SEM). All statistical analyses were performed using SPSS software, version 20.0 (SPSS Inc., Chicago, IL, USA). The differences between the means were analyzed using Student’s t test or one-way analysis of variance (ANOVA), followed by the Bonferroni post hoc analysis to determine the significance of specific comparisons. The differences were considered statistically significant at *p* < 0.05.

## 3. Results

### 3.1. Loss of Nsun5 causes CC Agenesis

The overall brain sizes of PND60 *Nsun5*-KO mice did not differ roughly from those of the littermate WT mice (Figure 1A). To investigate the possible role of Nsun5 in the development of CC, we performed Luxol Fast Blue (LFB) staining, a commonly used technique for detecting myelin sheaths [26], on the mid-sagittal CC and coronal CC sections from PND60 *Nsun5*-KO mice and wild-type (WT) mice (*n* = 6 per experimental group). As shown in Figure 1B,E the intensities of LFB staining in the mid-sagittal CC sections and the coronal CC sections were reduced in *Nsun5*-KO mice compared to WT littermates, indicating a decline in the myelin density. Notably, not only the intensity of LFB staining (*p* < 0.01; Figure 1C) but also the straight length of the mid-sagittal CC (*p* < 0.05; Figure 1D) in *Nsun5*-KO mice were less than those in WT mice. Subsequently, the coronal sections obtained from the rostral to caudal CC were divided equally into 7 segments (upper panels; Figure 1F) to measure the areas of the coronal CC (red dashed box) in different regions and the myelin density. In comparison with WT mice, the areas of the coronal CC in the 3rd–5th segments were significantly reduced in *Nsun5*-KO mice (*p* < 0.05; Figure 1H), while in other segments, there were no significant differences (*p* > 0.05). Similarly, the decline in the intensity of LFB staining was observed in the coronal CC of *Nsun5*-KO mice (2nd segment: *p* < 0.05; 3rd–5th segments: *p* < 0.01; Figure 1G). Although the intensity of LFB staining in other segments of *Nsun5*-KO mice had a decreasing tendency but the group when compared with WT mice failed to reach significance (*p* > 0.05). The results indicate that the loss of Nsun5 causes the volumetric reduction of CC with a decline of the myelin density.

### 3.2. Loss of Nsun5 Results in Myelination Defects of CC

**Figure 2:**
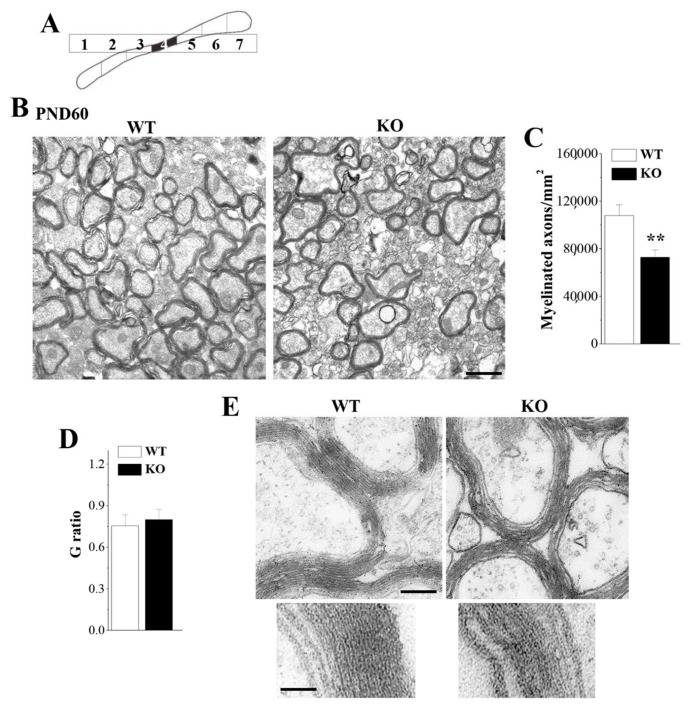
Loss of Nsun5 results in myelination defects in the CC. (**A**) Schematic diagram of coronal sections for TEM. (**B**) Representative transmission electron microscopy (TEM) images from 4th segment of CC (black region) in PND60 WT mice (WT) and *Nsun5*-KO mice (KO). Scale bar = 1 μm. (**C**) Number of myelinated axons in CC. ** *p* < 0.01 vs. WT (*n* = 6). (**D**) G ratio (numerical ratio of axonal diameter divided by the diameter of myelinated axons) in CC of PND60 WT mice and *Nsun5*-KO mice. (**E**) Representative higher power views of CC. Scale bar = 250 nm (upper); Scale bar = 100 nm (bottom).

To further determine whether the deletion of Nsun5 causes the ultrastructural alteration of callosal myelin, the cross-sections of CC (4th segment; Figure 2A) were examined by transmission electron microscopy (TEM, *n* = 6 per experimental group). As shown in Figure 2B. PND60 *Nsun5*-KO mice revealed structure turbulence and were missing the myelin sheath. The number of CC myelinated axons was significantly reduced in *Nsun5*-KO mice (*p* < 0.01; Figure 2C). Although G ratio analysis (the numerical ratio of the axonal diameter divided by the diameter of the myelinated axons) revealed that the myelin sheath thickness had no significant difference between the both groups (*p* > 0.05; Figure 2D), the myelination arrangement disorder and the loose myelin sheath were observed in the CC of *Nsun5*-KO mice (Figure 2E). The results indicate that the loss of Nsun5 leads to deficits in callosal myelination formation.

### 3.3. Nsun5 is Expressed in the OL Lineage of the Developing CC

**Figure 3:**
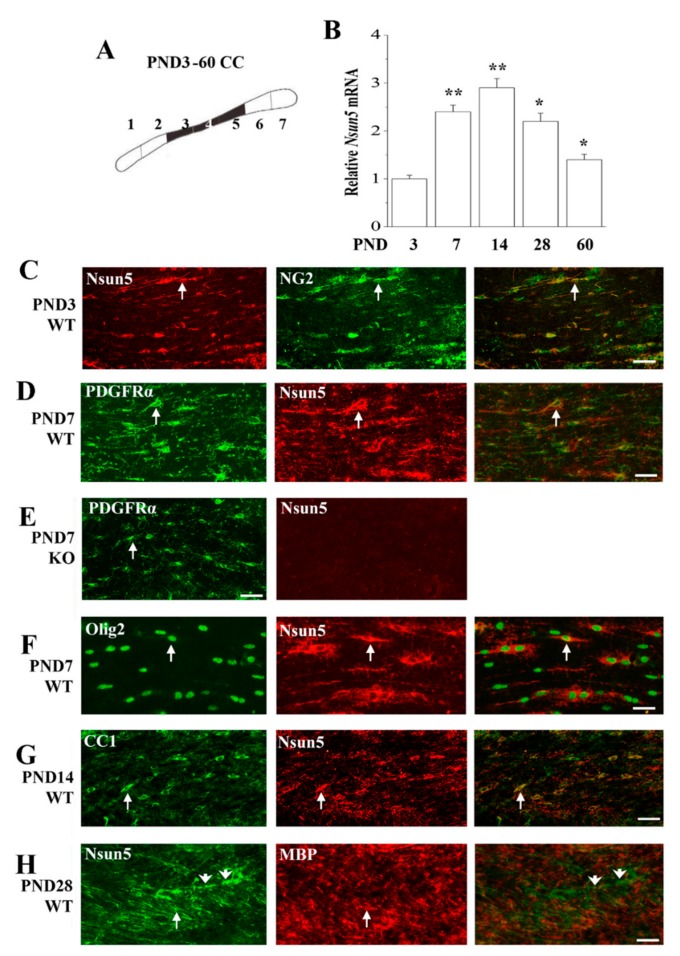
Nsun5 is expressed in oligodendrocyte (OL) lineage of developing CC. (**A**) Schematic diagram of CC obtained from wild-type (WT) mice. (**B**) Bar graphs show the levels of *Nsun5* mRNA obtained from 3rd to 5th segments (black region) of CC in PND3-60 WT mice. * *p* < 0.05 and ** *p* < 0.01 vs. PND3 mice (one-way ANOVA, *n* = 6). Representative images of double immunostaining for Nsun5 (green) and NG2 (red) in CC of PND3 WT mice, Scale bars = 30 μm (**C**); PDGFRα (green) and Nsun5 (red) in PND7 WT mice, Scale bars = 25 μm (**D**) and *Nsun5*-KO mice, Scale bars = 40 μm (**E**); Olig2 (green) and Nsun5 (red) in PND7 WT mice, Scale bars = 20 μm (**F**); CC1 (green) and Nsun5 (red) in PND14 WT mice, Scale bars = 25 μm (**G**); Nsun5 (green) and MBP (red) in PND28 WT mice. Scale bars = 30 μm (**H**).

During the development of the CC, the OPCs proliferate and differentiate to become mature OLs, generating myelin. To explore the mechanisms underlying the impaired myelination formation in *Nsun5*-KO mice, we first examined the dynamic level of Nsun5 expression in the CC of PND3-60 WT mice (Figure 3A, *n* = 6 per experimental group) since the proliferation and differentiation of OPCs on PND7-14 and the initiation of myelination on PND14-28 are the most active [15]. Real-time PCR analysis revealed the peak of Nsun5 expression between PND7-28 (*vs*. PND3, PND7-14: *p* < 0.01; PND28: *p* < 0.05; Figure 3B), which was well matched with the developmental period of the CC.

To further identify the characteristics of cells expressing Nsun5 during the development of the CC, we performed a double immunohistochemistry analysis by using Nsun5 with the OPC markers (NG2 and PDGFRα), the OL lineage marker (Olig2) and the mature OL markers (CC1 and MBP). On PND3, Nsun5 was expressed in NG2 positive (NG2+) OPCs (Figure 3C). The NG2+ OPCs revealed a biphasic pattern along the axon tracts of the CC. On PND7, we also found that Nsun5 could be detected in PDGFRα positive (PDGFRα+) OPCs (Figure 3D) and Olig2 positive (Olig2+) OLs (Figure 3F). Nsun5 resided in the cytoplasm and was involved in the processes of OPCs and OLs. This anti-Nsun5 antibody was highly specific, because no signal was detected in the CC of PND7 *Nsun5*-KO mice (Figure 3E). It is noteworthy that CC1-positive (CC1+) OLs on PND14 display Nsun5 positivity (Figure 3G). Consistently, the amount of Nsun5 protein in OPCs and OLs was progressively elevated from PND3 to PND7-14. On PND28, the Nsun5 protein was detected in the myelin sheath formed by mature OLs expressing MBP (Figure 3H). There were some Nsun5+/MBP- cells that showed the morphological features of OPCs (arrowhead), termed white matter OPCs in the CC. The expression of Nsun5 in the OPCs and mature myelinating OLs further supports the notion that Nsun5 can regulate the development of the CC.

### 3.4. Loss of Nsun5 Reduces OPCs and OLs Leading to Hypomyelination of CC

**Figure 4:**
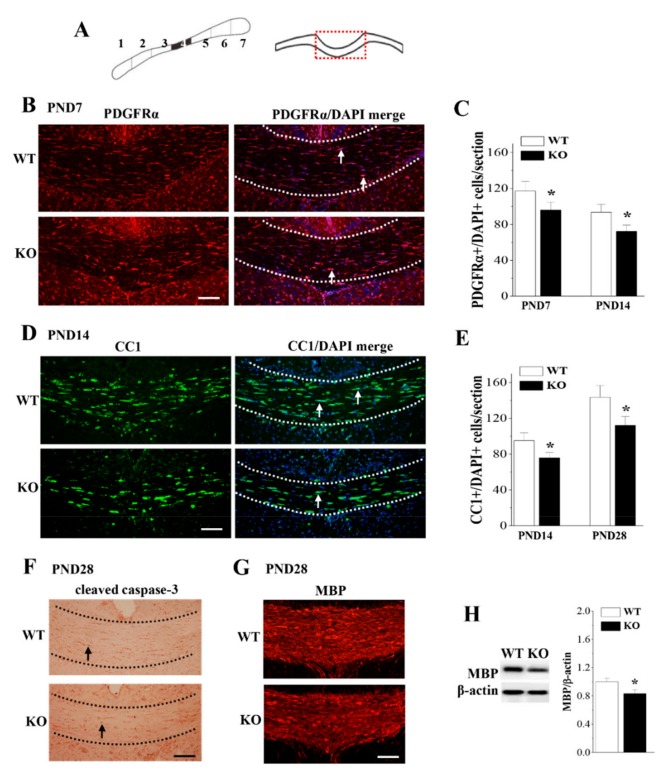
Loss of Nsun5 reduces OPCs and OLs leading to hypomyelination of CC. (**A**) Oligodendrocyte precursor cells (OPCs) and OLs were count in 4th segment of coronal CC (black region). (**B**) Representative images of immunohistochemistry for PDGFRα and PDGFRα/DAPI merge in PND7 WT mice (WT) and *Nsun5*-KO mice (KO). Scale bar = 35 μm. Arrows indicate PDGFRα+/DAPI+ cells. (**C**) Bar graph indicates the number of PDGFRα+/DAPI+ cells per section of CC (as shown in **A**; red dashed box) in PND7 and PND14 WT mice and *Nsun5*-KO mice. * *p* < 0.05 vs. WT (*n* = 6). (**D**) Representative images of immunohistochemistry for CC1 and CC1/DAPI merge in PND14 WT mice and *Nsun5*-KO mice. Scale bar = 25 μm. Arrows indicate CC1+/DAPI+ cells. (**E**) Bar graph indicates the number of CC1+/DAPI+ cells per section of CC in PND14 and PND28 WT mice and *Nsun5*-KO mice. * *p* < 0.05 vs. WT (*n* = 6). (**F**) Immunohistochemistry of caspase-3 in CC of PND28 WT mice and *Nsun5*-KO mice. Scale bars = 20 μm. (**G**) Representative images of immunohistochemistry for MBP. Scale bars = 20 μm. (**H**) The levels of MBP protein in CC of PND28 WT mice and *Nsun5*-KO mice. * *p* < 0.05 vs. WT (*n* = 6).

To further determine if the expression of Nsun5 is required for the proliferation and differentiation of OPCs, we quantified the total numbers of OPCs and OLs in the CC (red dashed box; Figure 4A) of PND7, PND14 or PND28 WT mice and *Nsun5*-KO mice (*n* = 6 mice per experimental group). As shown in Figure 4B, PDGFRα+ cells in the CC of PND7 *Nsun5*-KO mice was reduced. Quantitative measurement confirmed the decrease in the numbers of PDGFRα+/DAPI+ cells in PND7 (*p* < 0.05; Figure 4C) and PND14 (*p* < 0.05) *Nsun5*-KO mice compared to WT mice. We observed a significant reduction in the number of cells expressing the OL maturation marker CC1 in the CC of PND14 *Nsun5*-KO mice (Figure 4D). The numbers of CC1+/DAPI+ cells in PND14 (*p* < 0.05; Figure 4E) and PND28 (*p* < 0.05) *Nsun5*-KO mice were lower than those in WT mice. Next, we performed cleaved caspase-3 immunostaining to test whether the deletion of Nsun5 causes the apoptosis of OPC and OL. As shown in Figure 4F, the number of cleaved caspase-3 positive cells did not increase in PND28 *Nsun5*-KO mice. In addition, the hypomyelination in the CC of PND28 *Nsun5*-KO mice was observed by immunohistochemistry of myelin basic protein (MBP), a marker for mature OLs and myelin (Figure 4G). Furthermore, Western blot analysis showed an obvious reduction in the MBP protein level in *Nsun5*-KO mice compared with WT mice (*p* < 0.05; Figure 4H).

### 3.5. Loss of Nsun5 Suppresses the Proliferation of OPCs

**Figure 5:**
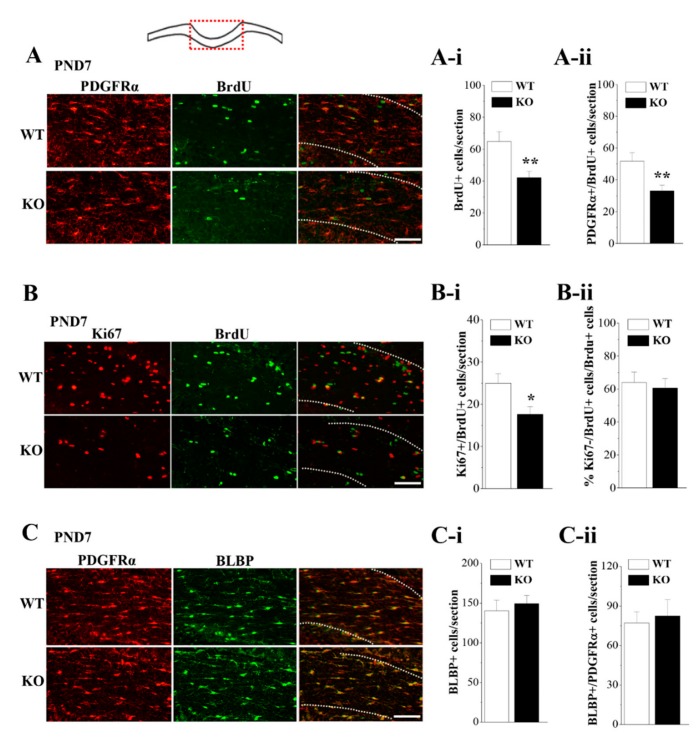
Nsun5 is required for the proliferation of OPCs. (**A**) Representative images of double immunostaining for PDGFRα (red) and Brdu (green) in CC of PND7 WT mice (WT) and *Nsun5*-KO mice (KO). Scale bar = 25 μm. Bar graphs indicate the number of BrdU+ cells (**A-i**) and PDGFRα +/BrdU+ cells (**A-ii**). ** *p* < 0.01 vs. WT (*n* = 6). (**B**) Representative images of double immunostaining for Ki67 (red) and BrdU (green) in PND7 WT mice and *Nsun5*-KO mice. Scale bar = 30 μm. Bars show the number of Ki67+/BrdU+ cells (**B-i**) and Ki67−/BrdU+ cells (**B-ii**). * *p* < 0.05 vs. WT (*n* = 6). (**C**) Representative images of double immunostaining for PDGFRα (red) and BLBP (green). Scale bar = 25 μm. Bars show the number of BLBP+ cells (**C-i**) and PDGFRα+/BrdU+ cells (**C-ii**).

To test whether the reduced OPCs in *Nsun5*-KO mice are due to deficits in OPC proliferation, we performed double immunostaining of PDGFRα with BrdU to label the proliferating OPCs in the CC of *Nsun5*-KO mice and their littermate controls (Figure 4A) (*n* = 6 mice per experimental group). We chose to examine PND7 CC based on our observation that Nsun5 was highly expressed and that OPCs were decreased in *Nsun5*-KO mice at this stage. Two hours after BrdU injection, the number of BrdU-positive (BrdU+) cells in *Nsun5*-KO mice was reduced by approximately 35% compared to the number in WT mice (*p* < 0.01; Figure 5A-i). In particular, the number of PDGFRα+/BrdU+ cells in *Nsun5*-KO mice showed an approximately 42% decline (*p* < 0.01; Figure 5A-ii), indicating that Nsun5 deficiency suppresses OPC proliferation.

BrdU is known to label a cohort of cells in the S phase, while Ki67 is expressed in proliferating cells throughout all phases of the cell cycle. Thus, the BrdU+/Ki67+ cells are thought to represent the proportion of cycling cells, whereas the remaining BrdU+/Ki67− cells exit from the cell cycle. To examine the cell cycle dynamics of proliferating OPCs, a cell cycle exit experiment was performed by quantifying the proportion of BrdU+/Ki67+ cells (*n* = 6 mice per experimental group). In comparison with that in WT mice, the number of Ki67+/BrdU+ cells was reduced in *Nsun5*-KO mice (*p* < 0.05; Figure 5B-i). However, the cell cycle exit index of OPCs (BrdU+/Ki67− cells divided by the total population of BrdU+ cells) in the CC of *Nsun5*-KO mice did not differ from that in WT mice (*p* > 0.05; Figure 5B-ii).

To exclude whether the reduced BrdU+ cells are due to the reduced transition of radial glial cells (RGCs) into OPCs, immunostaining of brain lipid-binding protein (BLBP), a reliable marker of RGC, was used. The number of BLBP+ cells (*p* > 0.05; Figure 5C-i) or BLBP+/PDGFRα+ cells (*p* > 0.05; Figure 5C-ii) in *Nsun5*-KO mice was unchanged compared to those in WT mice.

### 3.6. Loss of Nsun5 Suppresses CDK1/2 Expression

**Figure 6:**
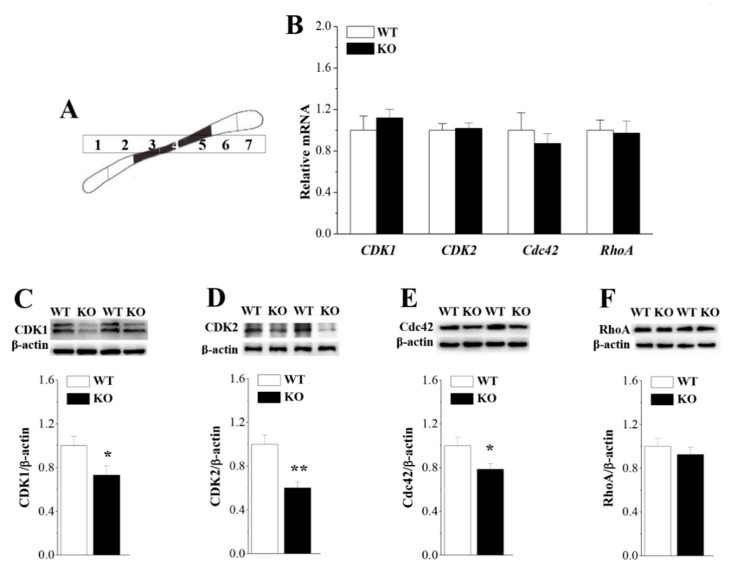
Loss of Nsun5 suppresses CDK1/2 and Cdc42 expression. (**A**) Schematic diagram of CC obtained from WT mice and *Nsun5*-KO mice. (**B**) Bar graphs show the levels of the *CDK1*, *CDK2*, *Cdc42* and *RhoA* mRNA in CC of WT mice (WT) and *Nsun5*-KO mice (KO). Bars represent the levels of the CDK1 (**C**), CDK2 (**D**), Cdc42 (**E**) and RhoA proteins (**F**) in CC of WT mice and *Nsun5*-KO mice. * *p* < 0.05, ** *p* < 0.01 vs. WT (*n* = 6).

Cyclin-dependent kinase 1 (CDK1) and CDK2 have been demonstrated to regulate cell proliferation [27]. CDK2 controls OPC cell cycle progression [27,28]. To further explore the mechanisms underlying the reduced proliferation of OPCs in *Nsun5*-KO mice, we examined the expression levels of CDK1 and CDK2 in isolated PND7 CC (Figure 6A, *n* = 6 mice per experimental group). The levels of *CDK1* (*p* > 0.05; Figure 6B) and *CDK2* mRNA (*p* > 0.05) in the CC of *Nsun5*-KO mice failed to be altered compared to those in WT mice. Interestingly, the western blot analysis showed a notable reduction in the level of the CDK1 protein (*p* < 0.05; Figure 6C) and the CDK2 protein (*p* < 0.01; Figure 6D).

Rho family GTPases, Cdc42 and RhoA, play an essential role in controlling the differentiation and processes of OLs [29]. The levels of *Cdc42* and *RhoA* mRNA in PND14 *Nsun5*-KO mice did not differ from that in WT mice (*p* > 0.05). The level of Cdc42 protein was lower in the CC of PND14 *Nsun5*-KO mice than it was in WT mice (*p* < 0.05; Figure 6E), whereas the RhoA protein did not decrease in *Nsun5*-KO mice (*p* > 0.05; Figure 6F).

## 4. Discussion

In the current study, we provided the first in vivo evidence that the loss of Nsun5 results in the agenesis of CC with postnatal hypomyelination of axons. This conclusion is deduced mainly from the following observations: the deletion of Nsun5 caused a decrease in the length of the sagittal CC (midline) and the area of the coronal CC (mid-segments), which was associated with fewer myelinated axons and the loose myelin sheath.

Nsun5 was highly expressed in OPCs during CC development. Notably, the number of OPCs (PDGFRα+ cells) was reduced in the CC of PND7 and PND14 *Nsun5*-KO mice. The size of the progenitor pool (the number of BLBP+ RGCs and BLBP+/PDGFRα+ cells) was unchanged in PND7 *Nsun5*-KO mice. Consistent with the report by Zhang et al. [14], the number of BrdU+/PDGFRα+ cells was lower in the CC of PND7 *Nsun5*-KO mice, indicating that the loss of Nsun5 suppresses the proliferation of OPCs. A principal finding in this study is that the expression of CDK1 and CDK2 was down-regulated in the CC of PND7 *Nsun5*-KO mice. The CDK2 activity has been reported to play a pivotal role in OPC cell cycle decisions occurring at G1/S checkpoint [28]. During the early G1 phase of proliferation, the pairing of CDK2 with cyclin E promotes entry into the S phase of the cell division cycle [30]; then CDK2 switches to partner with cyclin A to drive the cell though S phase [31]. CDK2 deletion in mouse embryo fibroblasts causes a delay in S phase entry [32]. The CDK1-cyclin B complex is thought to regulate the G2-M transition and progression through mitosis [33]. Although there are no reports of CDK1 mutant mice, mice lacking cyclin B2 do not show OPC cell cycle defects [34]. Thus, it is proposed that the Nsun5 deficiency may impede the S phase entry of OPCs [35]. The exclusion of exon 5 from the CDK2 transcription dramatically represses the expression of the CDK2 protein with a corresponding perturbation in cell cycle kinetics [36]. Caillava et al. [37], however, reported that the OPC proliferation in the CC during the early postnatal stages is CDK2-independent and that the CDK2 deficiency enhances the cycle exit of premature OPCs. Inconsistently, we observed that the proportion of exited cell cycle (percentage of Ki67−/BrdU+ cells against total BrdU+ cells) in PND7 *Nsun5*-KO mice was unchanged, indicating that the reduced proliferation of OPCs do not seem to be due to the prematurely exited cell cycle of OPCs. In Cdk2-deficient mice, CDK1 was found to compensate for the loss of CDK2 function by binding to cyclin E and regulating the G1/S transition [38]. Thus, one possible explanation is that the decline of CDK1/2 expression in *Nsun5*-KO mice is responsible for the reduced OPC proliferation.

Interestingly, the decrease in the CDK1/2 proteins in *Nsun5*-KO mice was not associated with transcription level changes in the *CDK1/2* mRNA. Nsun2, a tRNA methyltransferase, induces the methylation of tRNA to stabilize the tRNA and promote the protein synthesis [39]. An earlier study reported that the expression level of Nsun2 is cell cycle-dependent, with the highest expression in the S phase [40]. Nsun2 enhances CDK1 translation by methylating the CDK1 3′UTR at C1733 (m5C) without altering the *CDK1* mRNA level [41]. Sharma et al. [11] and Gigova et al. [12] have found that Rcm1—the yeast homologue of Nsun5—directly methylates 25S rRNA at cytosine 2278. Nsun5 ablation alters the structural conformation of the ribosome and the translational fidelity [13]. The Nsun5 deficiency is highly likely to affect the CDK1/2 expression at the translational level. Therefore, additional experiments will be needed to fully elucidate the mechanisms underlying the Nsun5 deletion-reduced CDK1/2 expression.

Notably, the number of OLs (CC1+ cells) was obviously reduced in the CC of PND14-28 *Nsun5*-KO mice. The OLs highly expressed Nsun5 during CC development, thus strongly indicating that Nsun5 is required for generating effective numbers of OLs. The apoptotic cells were not increased in the CC of PND28 *Nsun5*-KO mice, indicating that Nsun5 insufficiency does not impair OL survival. The OPCs generate the majority of myelinating OLs during the early postnatal period [42]. Although we cannot rule out the possibility that the deletion of Nsun5 may alter OPC differentiation, the reduced proliferation of OPC may be responsible for the decline of OLs.

We observed an obvious decline in the myelinated axons and hypomyelination in the CC of PND60 *Nsun5*-KO mice. Myelination occurs in a stepwise process where the OPCs proliferate and mature to become functional myelinating OLs. Olig2 ablation caused a nearly complete absence of myelination in the cortex during the early postnatal stages and severe dysmyelination, even in adulthood [43,44], suggesting that OPCs are a critical source for OL myelination in the developing cortex. On the other hand, Nsun5 is expressed in the process of mature OLs, implying its possible role in myelinogenesis. Our experimental data support this idea, because the deletion of Nsun5 was found to not only reduce the level of the MBP protein but also cause the myelination arrangement disorder and the loose myelin sheath in PND60 CC. Importantly, the expression level of Cdc42 was lower in the CC of PND14 *Nsun5*-KO mice, while no decrease was observed in the expression level of RhoA. Cdc42 activity is important for myelination when the OL processes ensheath the axons to form compacted myelin sheaths [45]. Cdc42 is thought to act as a positive regulator of OL process extension and branching [46]. The ablation of Cdc42 does not affect OPC proliferation or differentiation but it does lead to the extraordinary enlargement of the OL process and the abnormal accumulation of cytoplasmin [47]. Furthermore, the ablation of Cdc42 causes the widespread formation of aberrant myelin outfoldings through the abnormal accumulation of cytoplasm in the inner tongue of the oligodendrocyte process.

## 5. Conclusions

Pober reported that the *Nsun5* gene, a member of the NOL1/Nop2/sun protein family, is deleted in approximately 95% of patients with WBS. In this study, we determined that the loss of Nsun5 leads to the agenesis of CC with the postnatal hypomyelination of axons. The abnormal patterns of CC morphology and shape in the *Nsun5*-KO mice were similar to those in WBS individuals. The formation of myelin provides essential signals for the growth of axons as well as long-term structural integrity [48]. The agenesis of CC in *Nsun5*-KO mice suggests that the deletion of Nsun5 in WBS may play an important role in CC axonal growth and function. The fibers of the CC are implicated in a wide range of cognitive abilities [5,49]. Of note, the cognitive functions have been found to be affected in WBS [50]. Nsun5 is heterozygous in WBS patients [51]. *Nsun5*-KO mice and heterozygous *Nsun5* mice show the cognitive disorder phenotype [14], which is companied by the CC agenesis and hypomyelination (in this study). Although the single-gene knockout phenotype observed in the *Nsun5*-KO mice may not be relevant to the human deletion syndrome at all, these observed structural alterations in the CC might be associated with the cognitive behavioral profile of WBS patients.

## Figures and Tables

**Table 1:** Primers for quantitative real-time polymerase chain reaction (PCR).

Gene	Forward	Reverse
*Nsun5*	GAGGGAAGGGTGGATAAGG	GGCACGATGCGGATGTAG
*CDK2*	GTTGGTGATGGTGCTGTTG	CTGTGGATAACTTAGCGGTCG
*CDK1*	AAAGCGAGGAAGAAGGAG	GGACAGGAACTCAAAGATGA
*Cdc42*	GTTGGTGATGGTGCTGTTG	CTGTGGATAACTTAGCGGTCG
*RhoA*	CATTGACAGCCCTGATAGTT	TCGTCATTCCGAAGGTCCTT
*GAPDH*	TGGGTGTGAACCACGAG	ACCACAGTCCATGCCATCAC
